# Draft Genome Sequence of Lactococcus sp. Strain NtB2 (JCM 32569), Isolated from the Gut of the Higher Termite Nasutitermes takasagoensis

**DOI:** 10.1128/genomeA.00445-18

**Published:** 2018-06-14

**Authors:** Satoko Noda, Chihiro Aihara, Masahiro Yuki, Moriya Ohkuma

**Affiliations:** a Graduate School of Life and Environmental Sciences, University of Yamanashi, Kofu, Yamanashi, Japan; b Japan Collection of Microorganisms (JCM), RIKEN BioResource Research Center, Tsukuba, Ibaraki, Japan

## Abstract

Lactic acid bacteria are widely distributed in the termite gut. Here, we report the draft genome sequence of Lactococcus sp. strain NtB2, which was isolated from the gut of a wood-feeding higher termite.

## GENOME ANNOUNCEMENT

The relationship between termites and the microorganisms inhabiting their gut is one of the most remarkable examples of symbiosis. The relationship enables termites to feed on lignocellulose, and the microbes supply nutrients. Bacteria of the genus Lactococcus are widely distributed in not only the digestive tract of animals but also fermented foods. They have been reported to be one of the most important lactic acid bacteria in the hindgut of wood-feeding termites. Actually, several lactic acid bacteria, including Lactococcus species that have unique phenotypic features, have been isolated and described as new species (1–3). We isolated a bacterial strain belonging to the genus Lactococcus, designated strain NtB2 (deposited to the Japan Collection of Microorganisms [JCM] as JCM 32569), from the gut content of a wood-feeding higher termite, Nasutitermes takasagoensis.

The genome sequencing of Lactococcus sp. strain NtB2 was performed on the MiSeq platform (Illumina) using a MiSeq version 3 reagent kit (600 cycles). The generated reads were assembled using SPAdes version 3.11.1 (4). A total of 991,748 reads were assembled into 22 contigs with an *N*_50_ value of 181,959 bp and a largest length of 269,516 bp. This assembly resulted in a draft genome sequence of 1,734,781 bp with a G+C content of 47.5%. Totals of 1,667 protein-coding DNA sequences and 45 tRNAs were detected by Prokka (5) and the Rapid Annotations using Subsystems Technology (RAST) server (6).

Despite the presence of lactic acid bacteria in the hindgut of most termite species, lactate is not a major metabolite in the hindgut fluid (7). This discrepancy might be explained by a shift in the fermentation balance of lactic acid bacteria from lactate toward acetate formation in the presence of oxygen, as has been demonstrated for an Enterococcus sp. isolated from the wood-feeding lower termite Reticulitermes flavipes (7). It has been suggested that lactococci isolated from termite guts play a role in the fermentation of the degradation products of cellulose and hemicellulose, in oxygen reduction (7), and in the supply of folate required by acetogenic treponemes in the gut (8). The genome of strain NtB2 contains the genes involved in the biosynthesis of folate, namely, *folB*, *folE*, *folP*, *folC*, and *folA*. Although lactic acid bacteria are physiologically adapted to the termite gut environment, their impact on the symbiotic metabolism, such as carbon and electron fluxes, needs to be elucidated.

### Accession number(s).

The genome sequence of Lactococcus sp. strain NtB2 (JCM 32569) has been deposited at DDBJ/GenBank under the accession numbers BFFO01000001 to BFFO01000022.
